# Antimicrobial Properties of Colostrum and Milk

**DOI:** 10.3390/antibiotics13030251

**Published:** 2024-03-11

**Authors:** Furkan Eker, Emir Akdaşçi, Hatice Duman, Yalçın Mert Yalçıntaş, Ahmet Alperen Canbolat, Arda Erkan Kalkan, Sercan Karav, Dunja Šamec

**Affiliations:** 1 Department of Molecular Biology and Genetics, Çanakkale Onsekiz Mart University, Çanakkale 17000, Turkey; furkanekeerrr@gmail.com (F.E.); emirakdasci@gmail.com (E.A.); hhaticeduman@gmail.com (H.D.); yalcintasmert@gmail.com (Y.M.Y.); canbolatahmetalperen@gmail.com (A.A.C.); ardaerkankalkan@gmail.com (A.E.K.); 2 Department of Food Technology, University North, Trg Dr. Žarka Dolinara 1, 48000 Koprivnica, Croatia; dsamec@unin.hr

**Keywords:** milk, colostrum, immunity, bioactive components, antibacterial activity

## Abstract

The growing number of antibiotic resistance genes is putting a strain on the ecosystem and harming human health. In addition, consumers have developed a cautious attitude towards chemical preservatives. Colostrum and milk are excellent sources of antibacterial components that help to strengthen the immunity of the offspring and accelerate the maturation of the immune system. It is possible to study these important defenses of milk and colostrum, such as lactoferrin, lysozyme, immunoglobulins, oligosaccharides, etc., as biotherapeutic agents for the prevention and treatment of numerous infections caused by microbes. Each of these components has different mechanisms and interactions in various places. The compound’s mechanisms of action determine where the antibacterial activity appears. The activation of the antibacterial activity of milk and colostrum compounds can start in the infant’s mouth during lactation and continue in the gastrointestinal regions. These antibacterial properties possess potential for therapeutic uses. In order to discover new perspectives and methods for the treatment of bacterial infections, additional investigations of the mechanisms of action and potential complexes are required.

## 1. Introduction

Milk is a nutritional secretion that is tailored to the needs of newborns and can be modified depending on the species [1]. For this reason, the composition of milk components may vary [2], and these variations may alter the effect of milk on the mechanism and efficacy of activity. The main components of milk consist mainly of whey, casein, fats, lactose, and plenty of water to meet the general needs of newborns [3]. In addition to the main nutrients, milk also contains immunomodulatory and antipathogenic proteins such as xanthine oxidase (XO), lactoperoxidase (LPO), immunoglobulins (Igs), lysozyme (LZ), lactoferrin (Lf), etc. [4]. However, the concentration of these components is significantly lower, and some of them are even so low that they show no effects after consumption.

The milk secreted by mammals during the first days, namely the first 48 h of lactation, is known as colostrum. Colostrum serves as the main source of nutrients necessary for the growth and development of the newborn [5]. It differs significantly from mature milk in its composition and nutrient content. It is very rich in many immune-related molecules and growth factors, such as Igs, Lf, oligosaccharides (OS), LZ, and epidermal growth factor (EGF) [6,7]. It should be particularly emphasized that colostrum contains a high concentration of IgG, which is an essential component for the passive immunity of newborns [8]. It also has a higher concentration of lipids, proteins, minerals, and vitamins compared to mature milk [9]. In addition, the composition and quality of colostrum can be influenced by various inherent and surrounding factors such as environment, breed, individual characteristics, milking time, and diseases [10]. All in all, colostrum can be considered a key component to promote neonatal growth, development, and immune defense [6]. In addition, colostrum, especially bovine colostrum, which contains significant amounts of certain bioactive compounds, has a greater potential to be used in a broader spectrum for the treatment of gastrointestinal disorders and diseases, as it prevents the growth of pathogenic microorganisms and promotes beneficial microbiota, among others [7,11].

In this article, we summarized information on the antimicrobial effects of colostrum and milk, with particular emphasis on the discussion of antibacterial compounds and their mechanisms of action.

## 2. Antibacterial Components of Milk/Colostrum

The antibacterial components and their content in milk/colostrum may vary depending on the source or other factors. The list of antibacterial compounds and their content in human and bovine milk and colostrum is shown in Table 1.

### 2.1. Lactoferrin

Lf is a multifunctional glycoprotein that is remarkably similar to the transferrin present in blood serum and has a considerable binding affinity for iron. Lf is widely distributed in a variety of mammalian secretions, such as the secondary granules of neutrophils, bronchial and intestinal secretions, tears, and milk [20]. Human breast milk has the highest concentration, followed by cow and buffalo milk. It is present in varying concentrations in the milk of different animal species. It is also the second most abundant protein in human milk, and colostrum has a higher content of it [21]. Bovine milk, on the other hand, contains less, namely 0.09 mg/mL in mature milk and 0.83 mg/mL in bovine colostrum. It is often referred to as a natural antibiotic and is an essential component that bridges the gap between the innate and adaptive immune systems of mammals and protects human cells for the duration of their lives [22,23]. In addition to promoting nutritional status and defense against microbial diseases, Lf has a variety of functions, including immunomodulatory, enzymatic, antioxidant, antiviral, antibacterial, and anti-inflammatory effects [24,25,26].

**Figure 1:**
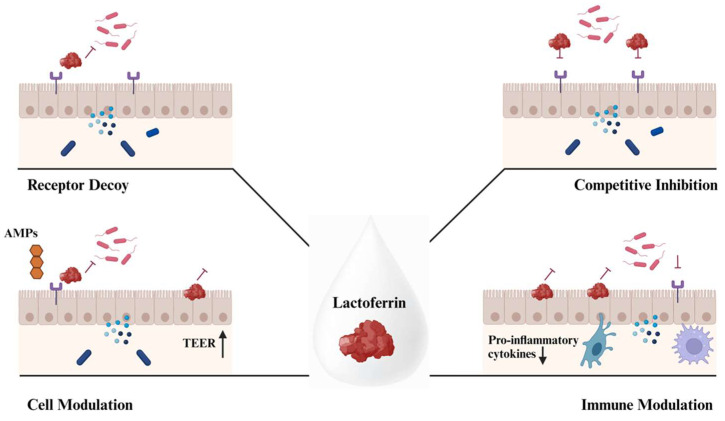
A schematic diagram describing the different modes of action of Lf to combat bacterial infections. Competitive inhibition occurs when Lf binds directly to the cell surface receptors, preventing the pathogen from attaching and blocking the first phase necessary for further colonization. Intestinal barrier modification occurs when Lf alters the surface of epithelial cells, triggering the release of antimicrobial peptides, modifying the carbohydrate components of membrane proteins, increasing the expression of tight junction proteins such as occludin, and strengthening the intestinal barrier. In terms of immunomodulation, Lf controls immunological responses by reducing uncontrolled pro-inflammatory responses that occur during infection, thereby averting damage to epithelial cells. In addition, Lf can bind to and activate phagocytic cells to induce phagocytosis of bacterial particles [33].

Lf can act in a variety of ways, including directly inhibiting or eliminating pathogens, stimulating or suppressing the immune system, or maintaining the intestinal epithelium via the formation of tight junction proteins. Since Lf binds to iron, its absence is also associated with a simultaneous interruption of bacterial growth, which prevents infection of the body [27]. In addition, it has an immunomodulatory effect, accelerating the development of immune system cells and stimulating the body to produce chemokines and cytokines (Figure 1) [28,29,30,31]. In addition, the bacterial cell membrane is damaged, or its metabolism is altered. The direct antibacterial effect of Lf is further emphasized by its ability to bind free iron and extract it from the microbial environment. By influencing the immune system in its defense against infections, Lf has an indirect effect (Figure 1) [32].

The protein sequences of human and bovine Lf are very similar (77%) [34,35]. As a result, bovine Lf has been used in many in vitro and in vivo studies (Table 2).

Currently, there are several thorough reviews that thoroughly investigate the anti-infective properties of Lf [51,52,53,54]. Lf is both bacteriostatic and bactericidal, as it kills a large number of pathogens while restricting the growth of several others. The most widely used type of Lf from human milk (hLf) is iron-free Lf, which has been shown to be effective against several bacteria, including *Pseudomonas aeruginosa*, *Candida albicans*, *Vibrio cholera*, *Streptococcus pneumoniae*, *Streptococcus mutans*, and *Escherichia coli* [27,55]. The ability of Lf to deprive bacteria of the iron they need to grow contributes to its bacteriostatic properties. Apart from its iron-chelating properties, it also exhibits antiviral, antifungal, and antiprotozoal properties [55]. Antimicrobial peptides (AMPs) generated from milk and colostrum play a significant role in the innate defense system, particularly on mucosal surfaces such as the small intestine and lungs that are continuously exposed to a variety of pathogens. Peptides with antibacterial properties are released by the proteolysis of Lf [56]. For example, lactoferricin, a peptide derivative of Lf, has been found to share several biological functions with Lf [57]. Similar to Lf antimicrobial mechanisms, the specificity of milk and colostrum-derived AMPs against specific organisms is maintained via various mechanisms, including targeted interactions, recognition of specific patterns and structures of pathogens, as well as evolutionary processes that enhance the specificity of AMPs against pathogens commonly encountered. The charge and hydrophobicity of AMPs are also important for the specificity of these peptides against pathogens. This structural characteristic enables them to specifically target and damage bacteria membranes [34]. Tossi et al., for example, found that raising the charge of the magainin-2 peptide from +2 to +5 enhanced its antibacterial effectiveness while maintaining the stability of other parameters. Additionally, it was noted that the antibacterial activity was not enhanced by the cationic charge increase from +6 to +7 [58]. Chen and colleagues showed that the L-V13K peptide’s enhanced hydrophobicity increased its efficacy against RBCs by a factor of 62.5. Peptide oligomerization or dimerization results from hydrophobicity increases above a particular threshold, and energetically stable peptide aggregates are the end product [59]. Together with the antimicrobial activity of AMPs derived from milk and colostrum, these bioactive peptides are also found in the gut microbiome and have important roles in biological processes. By fighting bacteria that are resistant to several antibiotics, gut AMPs work in concert with other gut microbiota and antimicrobials to preserve gut homeostasis. Moreover, the consumption of conventional antibiotics induces a synergistic evolutionary pressure on gut AMPs, wherein the two agents collaborate to combat multi-organ failure [60].

The observation that the administration of bovine Lf to extremely low birth weight neonates protects against necrotizing enterocolitis (NEC) and late-onset sepsis due to a variety of infections supports these preclinical findings [49,61,62]. Lf helps fight disease as it is effective against both Gram-positive and Gram-negative bacteria thanks to its antibacterial properties. Moreover, it is important in preventing harmful bacteria like *Staphylococcus aureus* and *P. aeruginosa* from forming biofilms [63,64]. For another instance, Kutila et al. [65] studied the antibacterial effects of Lf on a variety of udder bacterial isolates, which were initially obtained from bovine mastitis. It was discovered that Lf had the greatest inhibitory effect on *E. coli* and *P. aeruginosa*. The growth patterns of all five *E. coli* isolates were comparable. According to the study, Lf may have some antibacterial properties overall, particularly against *E. coli* and *P. aeruginosa*, albeit the degree of inhibition would depend on the concentration and growth medium. It was determined in a different study that Lf exhibited greater inhibitory qualities in Gram-positive bacteria than in Gram-negative bacteria due to its antimicrobial efficacy against both types of bacteria [66]. The study by Wang et al. [67] investigated and contrasted the antibacterial properties of deer Lf and its hydrolysates with those of its bovine counterpart. The deer Lf that had been digested in the duodenum and gastric showed potent bactericidal properties against *E. coli*, with minimum inhibitory concentrations (MIC) of 402 µM and 280 µM, respectively. These results suggest that bioactive whey proteins found in deer milk can produce peptides with antibacterial features, which may have a positive impact on health by preventing the growth of food-borne pathogenic bacteria. Similar research comparing the antimicrobial activities of deer and bovine lactoferricin and lactoferrampin against *E. coli* and *Lactobacillus acidophilus* examined the antibacterial activity of deer milk Lf and its hydrolates, including lactoferricin and lactoferrampin. The study’s findings showed that, according to MIC, deer lactoferricin was a more effective inhibitor of *L. acidophilus* than bovine lactoferricin, but that lactoferrampin and bovine lactoferricin were more effective against *E. coli* [68]. In another study investigating the antibacterial activity of different types of Lf, investigating Lf’s effects on both desiccated and non-desiccated *Cronobacter sakazakii* in diverse media was the goal of the study. With its activity increasing with concentration and time, native bLf was shown to be the more potent form in preventing *C. sakazakii* growth in all media based on the research results. The results of the study demonstrate that iron sequestration is the primary mechanism underlying bovine Lf’s (bLf) antibacterial action against *C. sakazakii*. Interestingly, bLf that was iron-saturated showed some effectiveness in lowering the viability of *C. sakazakii* in whey. The phosphate buffer-desiccated bacteria did not show increased sensitivity to native bLf. While natural bLf showed some antibacterial activity against desiccated cells in whey or milk, it was significantly less effective in these media against non-desiccated *C. sakazakii* than it was in phosphate buffer. The study also evaluated how heat treatments affected the antibacterial activity of native bLf. The findings indicated that treatments at 72 °C and 85 °C for 15 s, and 63 °C for 30 min completely conserved the activity against bacteria [69]. Similar research on Lf’s antibacterial activity and its effect on foodborne microorganisms was conducted. *Listeria monocytogenes* was tested by Ripolles et al. [70] for the activity against the bacteria of bovine whey fractions, pepsin, chymosin, and microbial rennet hydrolysates containing bLf. With the exception of chymosin and microbial rennet hydrolysates at low concentrations, the hydrolysates obtained with each enzyme were shown to be inhibitory of bacterial growth, albeit the activity was less than that of the whole Lf. In another study investigating the antibacterial properties of similar pathogens, hLf from milk, recombinant hLf (rhLf) from *Aspergillus awamori*, and their pepsin-derived hydrolysates were used. Except for *L. monocytogenes*, where rhLf showed higher activity, both hLf and rhLf behaved similarly in terms of MICs. Heat treatments had a significant effect on the antibacterial activity, except when 85 °C was applied for 10 min. Viable cell counts decreased in UHT milk and whey when hLf and rhLf were evaluated against *L. monocytogenes* and *E. coli* O157:H7, although not as much as in broth media [71].

In contrast to previous studies investigating the antibacterial activity of Lf, Conesa et al. [72] used ion-exchange chromatography to isolate Lf from the milk of a range of animals, including sheep, goats, camels, alpacas, elephants, and humans. The antimicrobial activity of the isolated Lf from different animals and humans against *E. coli* 0157:H7 was investigated using MIC and minimum bactericidal concentrations (MBCs). In contrast to alpacas and hLf’s, camel Lf showed the greatest efficacy against *E. coli* 0157:H7. Overall, the study shows differences between Lf’s from different animal sources in terms of their thermal stability and antibacterial activity.

Together with the antibacterial properties of Lf, a strong synergy between Lf and LZ in the destruction of bacterial membranes has been demonstrated [73]. In addition, Lf makes bacteria more susceptible to drugs such as vancomycin and penicillin [74]. In response to the increasing resistance of fungal and bacterial strains to conventional treatments, researchers are exploring novel therapeutic chemicals to improve the efficacy of current treatments. Promising findings from Lf research are opening new avenues for the use of Lf and its peptides to treat and prevent a variety of bacterial diseases. Also, these research findings have led to an increased interest in milk-derived AMPs as a safe and effective antibiotic substitute, with the added benefit of application in food to target shelf-life extension.

### 2.2. Lysozyme

**Figure 2:**
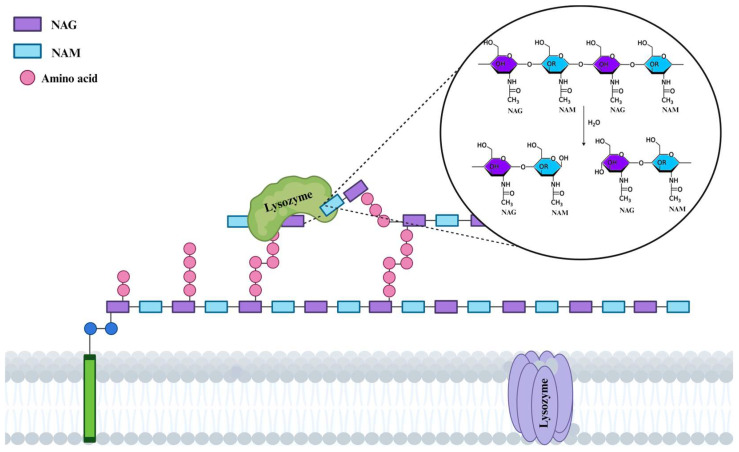
Demonstration of LZ showing enzymatic activity against bacterial cell wall. By cleaving the links between N-acetyl-D-glucosamine and ß-1,4-N-acetylmuramic acid in the peptidoglycan structure of bacterial cell walls, LZ demonstrates enzymatic activity [76].

LZ is an antibacterial protein with a single polypeptide chain containing 129 amino acids. It exhibits enzymatic activity by hydrolyzing bonds between ß-1,4-N-acetylmuramic acid and N-acetyl-D-glucosamine in the peptidoglycan of bacterial cell walls [75,76] (Figure 2). Due to its ability to degrade the bacterial cell wall, LZ is considered an endogenous antibiotic that is essential for the defense against harmful bacteria [77]. LZ, a component of the innate immune system, is present in a variety of secretions, including milk, mucus, tears, saliva, and urine [76]. Although the standard concentration of LZ in human milk is 200–400 µg/mL, it can reach ranges between 3 and 3000 µg/mL. On the other hand, cow’s milk contains only traces, about 0.05–0.22 µg/mL [17]. As the concentration of LZ in human colostrum and milk is high, it can be considered a valuable source for improving immunity and protecting human infants from microbial infections. In addition to human secretions, LZ can also be found in the organs and secretions of various mammals, microorganisms, and plants. In particular, chicken egg white, which contains about 0.3% LZ and accounts for 3.4–5.8% of the total protein content of egg white, is considered a significant source of LZ [77].

LZs are divided into three major families: chicken or conventional type (c-type), goose type (g-type), and invertebrate type (i-type) [78]. These LZs, primarily C-type, are widely used in scientific research. To give an example, a study aimed to observe the antibacterial activity of amyloid fibrils and oligomers formed from hen egg white LZ (HEWL) against *S. aureus* and *E*. *coli* [79]. Another following example analyzed the expression, activity, and antibacterial effect of C-type LZ by the characterization of LZ from *Scophthalmus maximus*, SmLysC [80]. The antibacterial activity of HEWL fibrils was enhanced against LZ-resistant *S. aureus* and LZ-insensitive *E. coli*, according to the results. The HEWL oligomers, in contrast, did not manage to show a considerable improvement in the antibacterial activity compared to native HEWL. Regarding the outcomes of the second research, purified recombinant SmLysC, which has the conserved E50 and D67 residues that form the putative catalytic site, exhibited bacteriolytic activity that resembles a catalytic mechanism when compared to higher vertebrate LZs.

Some features of LZ, including amino acid sequences, biochemical traits, and particularly enzymatic qualities, can vary based on the source and varieties [78]. The research was carried out to analyze the characteristic and enzymatic activity of the c-type LZ, found in crucian carp, against infectious *Aeromonas salmonicida,* indicating that recombinantly produced C-type LZ exhibiting significant in vitro antibacterial activity against *A. salmonicida*, with an average 0.92 cm radius inhibition zone, when 40 μg LZ is used [81]. In addition, research aiming to induce health benefits via secreting hLZ by using *Sacharomyces boulardii*, a probiotic yeast, investigated the changes in the gut microbiome and fecal metabolomes in mice after the introduction of engineered *S.boulardii*. It was concluded that *S. boulardii* is able to increase beneficial microbial diversity and change the gut microbiome structure by promoting the growth and diversity of beneficial clostridia [37] Table 1. Also, various factors such as osmotic strength, pH, salt concentration, and temperature can affect the activity of LZ [75]. LZ exhibits its highest antibacterial activity at a pH of approximately 5. Throughout the investigation, HEWL was exposed to heat stress at pH 3.0–7.0 between 25 °C and 95 °C, with FTIR spectroscopy being used to track the samples. The enzyme had the highest degree of thermal stability at pH 5.0, as determined by calculated Tm values in comparison to other pH values [82]. Another research aimed to investigate the effect of thermal treatments such as pasteurization and condensation on the antibacterial activity of LZ in jenny milk. In the course of the research, they evaluated the antibacterial activity of jenny milk against the following bacteria: *E. coli*, *Xanthomonas campestris*, *Clavibacter michiganensis*, *Bacillus megaterium*, and *Bacillus mojavensis.* The results indicated that condensation and pasteurization retained most jenny milk’s antibacterial properties except for *B. mojaventis*. Specifically, LZ in jenny milk showed synthetic antibiotic-like antibacterial action against *C. michiganensis* and *X. campestris*. Additionally, it was also observed that the concentration of the LZ remained unchanged after thermal treatments [83].

Compositionally, LZ is a compact globular enzyme with a deep groove on the protein surface. It is an active region that helps the LZ to attach the peptidoglycan structure of bacterial cell wall to hydrolyze ß-1,4-N-acetylmuramic acid (NAM) and N-Acetyl-D-glucosamine (NAG) linkages [76,84] (Figure 2). Based on Fleming’s findings, the lytic activity of LZ has historically been determined by monitoring the lysis of UV-killed and lyophilized *Micrococcus luteus* cells. Shugar carried out the following modifications [85]. More recently, it has been used in protein purification, DNA extraction, RNA extraction, and laboratory techniques such as Northern blotting to lyse cells in bacterial expression systems. Additionally, peptides derived from LZ are also able to show lytic activity. A study was carried out to examine the antibacterial role of the peptides of human milk LZ (hLZ). Pepsin was used to generate five different peptide motifs, helix-loop-helix (HLH), two helices (H1 and H2), and two helix-sheets (H2-S12 and H2-S13) from hLZ. Each of the peptide motifs derived from hLZ was characterized and examined for antibacterial activity. HLH peptide and its N-terminal helix, among others, were found to have considerable bactericidal activity against Gram-positive, Gram-negative bacteria and the fungus *C. albicans*. Another study conducted by the same author examined the antimicrobial potential of the HLH peptide [86]. It was highlighted that 16 out of 28 amino acid sequences of the HLH domain of hLZ and c-type LZ were identical. Due to this level of similarity, it is assumed that HLH peptide might play a crucial role in revealing the antimicrobial activity of LZ. Additionally, the HLH peptide’s ability to disrupt microbial membranes via channel formation and self-promoted uptake would represent a promising direction for the development of new antimicrobial drugs. Consequently, it was pointed out that hLZ having multiple antibacterial peptide motifs would provide insight into new potential uses in the long run [87]. For instance, LZ has been tested as an efficiency enhancer for antibiotic treatment. In a study investigating the administration of antibiotics and LZ, both together and separately, on *Pseudomonas aeroginosa* [88]. To identify if the combination of LZ can enhance antibiotic efficiency, ceftazidime and cefapime were tested during the experiment. The results showed that using these antibiotics combined with LZ significantly increased the reduction in biofilm mass in the average value of tested 16 isolates when compared with solo antibiotic treatment. On the other hand, the usage of LZ as an alternative antibiotic has also been discussed [77]. It has been emphasized that *S. aureus*, a Gram-positive bacteria responsible for numerous types of clinical illnesses, is a threatening agent for skin infections and is known to show antimicrobial resistance (AMR). LZ has been tested in mice to test its replaceability for antibiotics. It was indicated that results obtained from LZ activity were enough to compare it as an alternative for synthetic antibiotic treatment in this case. When all considered, LZ, with the configuration mentioned earlier, demonstrates antibacterial activity against a wide variety of bacteria and inhibits viruses, parasites, and fungi [89].

In conclusion, when multiple biochemical and immunological properties of LZ are considered, despite having less concentration when compared to other milk antibacterial components such as Igs and Lf, it can be regarded as a significant antibacterial agent with a unique capability to hydrolyze NAM and NAG linkages in the bacterial cell wall. Although LZ studies are currently limited to enhancement applications, conducting research at a molecular level will create a new working perspective to milk antibacterial mechanisms in the future.

### 2.3. Xanthine Oxidase

**Figure 3:**
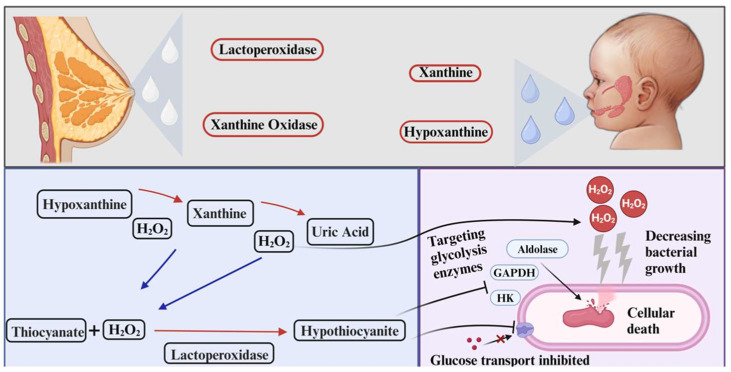
Scheme of XO-LPO systems with their combined antibacterial activity. Breast milk XO can use the substrates found in the neonatal saliva during lactation to initiate the uric acid pathway that creates H_2_O_2_ in the process. Excess H_2_O_2_ can be involve in the LPOS if the system has the required components. Alternatively, produced H_2_O_2_ can directly show antibacterial activity as disrupting bacterial growth. Thereafter, milk LPO creates the antibacterial molecule hypothiocyanite to inhibit bacterial enzymes and transport proteins that cause cellular death of the bacteria [100].

XO is a form of xanthine oxidoreductase (XOR) and can be bi-directionally converted to xanthine dehydrogenase (XDH) based on the presence of specific substrates [90]. When oxygen is accessible as a substrate, XO catalyzes the oxidation of xanthine (and/or hypoxanthine to xanthine) to uric acid by generating a superoxide molecule [91]. The catalysis reaction generally alters immune responses and oxidative stress levels, which is mostly likely due to the increase in uric acid concentrations [92]. There are contrary results of uric acid research as some studies indicate uric acid can show antioxidant activity in certain environmental conditions or as a pro-oxidant molecule and contribute to oxidative stress, especially in accumulated amounts [93]. XOR can be altered with post-translational modifications to XO and has a role in purine metabolism for uric acid synthesis in several tissues [94]. Distinctly, XOR also plays a role in lactation and pregnancy in mammals and is the major protein component in the milk fat globule membrane [95]. Expressed XO in breast tissue produces hydrogen peroxide (H_2_O_2_) and nitric oxide (NO), which is followed by the utilization of these molecules by LPO that leads an environment in the breast to prevent bacterial growth (which will be discussed in the further section) (Figure 3) [96]. To point out the substantial part, XO has been investigated as an antibacterial enzyme for a long time. Therefore, XO isolation and activity in milk under certain conditions have been investigated and discussed [97,98]. For instance, research investigated the kinetic aspects of inactivation and antibacterial activity of XO against several heat treatment methods on milk [91]. For the antibacterial activity test, *S. aureus* was used for XO activity after the heat treatments. The experiment showed the XO’s antibacterial activity, and its ability to preserve its activity against heat treatment methods (except ultra-high temperature).

The XO-LPO system has been referred to in a study that characterizes milk components as producing oxygen species to generate antimicrobial activity and protect the mammalian gland against bacterial infection [98]. The same system is also referred to by the same author in another research that directly investigated bovine milk XO’s antimicrobial activity [99]. The antibacterial activity of bovine XO by H_2_O_2_ production against *S. aureus* inhibits its growth, depending on the dose concentration. The antibacterial activity was tested with the addition of certain substrates, such as hypoxanthine and xanthine, to the bovine milk. The research confirmed the antibacterial activity belonged to the XO by showing the disappearance of the activity when the XO inhibitors were administered. Finally, in dose-dependent manners, *E. coli* O157:H7, *K. pneumoniae*, and *L. monocytogenes* were also inhibited in the bovine milk when certain amounts of hypoxanthine (50, 100, and 200 µM, respectively) were introduced. Only the highest concentration (400 µM) managed to inhibit *Enterococcus faecalis* by 60%. The rate activity of XO can also be different based on the source. For instance, research investigated the structure and functional aspects of XOR to identify the antibacterial activity of both buffalo and cattle XOR, as cattle XOR showed higher activity in higher doses when compared [95]. The ability to generate reactive oxygen species (ROS) comprises antibacterial activity, potentially creating both bactericidal and bacteriostatic characteristics. The bactericidal activity of XO was investigated in detail by a research group in breast milk. The research group investigated the activity of XO in breast milk by combining it with neonatal saliva after the analysis of neonatal saliva showed a high concentration of XO substrates, xanthine, and hypoxanthine [100]. The incubated bacteria, *S. aureus*, *Lactobacillus plantarum*, and *Salmonella* spp. showed a significant decrease in growth on the exposure of H_2_O_2_ generated by the milk-saliva mixture. The antibacterial activity of XO was confirmed as the addition of an inhibitor of the XO removed the bactericidal effect. It has been thought that the activation of H_2_O_2_ production by XO from neonatal saliva substrates is normally initiated in the lactation of the infant. To investigate further, the same research group investigated the activity of XO in the same setup but increased the width of microorganisms in more detail [101]. The microbial growth of certain microorganisms was investigated under the exposure of breastmilk and neonatal saliva, just like the previous research. The in vitro study on a similar in vivo environment of an infant’s mouth was designed and a wide range of microorganism species’ growth was inhibited. The antibacterial activity was also correlated with XO with XO inhibitor addition. The effect of using multiple bacteria at once was also tested to see if either one of them grew dominantly over the other organism. The results showed that each matched two bacteria species could not grow dominantly on the other in the in vitro system. They stated the general mechanism behind the antibacterial activity as the synthesis of ROS by breastmilk, followed by the initiation of the LPO system to decrease bacteria growth.

As discussed, it can be observed that XO has a strong and wide range of antibacterial activity that can produce ROS and disrupt bacterial growth. The fact that XO can be mainly found in milk is an important factor in antibacterial activity. The most highlighted study in this section pointed out the relationship of XO and its antibacterial activity between breast milk and newborns. The feature of neonatal saliva by containing substrates for XO creates a natural interaction between breast milk XO during lactation, thus potentially indicating the formation of XO’s antibacterial activity according to the evolutionary necessity.

The impact of the relationship between the mother and the child is not only limited by the XO and LPO interaction. The unique microbiome of human milk and its components are the primary source in the development of an infant’s gut microbiome and the XO-LPO system is just one of the regulators. The reason why this system is highlighted up to this point of the review is to point out that the antimicrobial activity of milk compounds is initiated immediately during lactation with a unique combination. In terms of gut modulation, it can be observed that other milk compounds, both antimicrobial and non-antimicrobial, can also regulate the shape of the gut microbiome and alter it favorably to the newborn. For instance, human milk OS (HMOs) are mainly known for their prebiotic factors for bifidobacteria in the infant intestine [102]. One of the unique characteristics of HMOs is that they can specifically increase the growth rate of bifidobacteria, increasing their dominance in the infant microbiome and creating pressure on pathogenic bacteria with bacterial competition [103]. The selective interaction of HMOs with pathogenic bacteria, which will be discussed in the further section of this review, is also shifting the favorability into the beneficial bacteria in the microbiome. Similar activity is also observed in IgA. IgA is the most abundant type of Ig that is synthesized from the mucosal surface. IgA is highly found in the human colostrum and plays a crucial role in the development and maturation of the infant gut microbiome development and maturation of the infant gut microbiome [104]. The influence of IgA on microbial diversity is rather less impactful when compared with its effect on supporting the dominance of beneficial bacteria in the gut. This is most probably caused by the similarity between IgA and IgM, as the influence on microbial diversity will not be solely dependent on a single type of Ig. This similarity was discussed in a study. The influence of IgA on microbial diversity is rather less impactful when compared with its effect on supporting the dominance of beneficial bacteria in the gut. This is most probably caused by the similarity between IgA and IgM, as the influence on microbial diversity will not be solely dependent on a single type of Ig. This similarity was discussed in a study [105] by indicating the potential similarity between the mechanisms of IgA and IgM that leads to influencing the same microbial populations. Since certain abnormalities in the gut and microbiome can be observed in the absence or insufficient levels of IgA, it is thought that IgA potentially controls some populations of bacteria in the gut in beneficial manners via several mechanisms [104]. To reference the previous paragraph, the evolutionary necessity is also influencing the other antibacterial compounds of milk by being in high amounts in colostrum from all mammals. These compounds are not only equipped with their antibacterial ability to control pathogenic populations but are also capable of increasing the selectivity and abundance of beneficial bacteria.

### 2.4. Lactoperoxidase

LPO is an enzyme in the group of peroxidases that is mainly found in the mammary gland to function as a protective agent on glands and milk [106]. LPO can increase the efficiency of the production of antibacterial molecules, along with its main function of catalyzing the oxidation of molecules when H_2_O_2_ exists in the environment [107]. To achieve a more comprehensive mechanism and activity, LPO forms the LPO system (LPOS) with thiocyanate (SCN^−^) and H_2_O_2_ [106]. The system initiates the oxidation of SCN^−^ by LPO with H_2_O_2_ to create a hypothiocyanite ion, an effective antimicrobial molecule [108]. Hypothiocyanite can target -SH groups, partially specifically, and inhibit certain vital metabolic pathways (certain glycolysis enzymes) and transport mechanisms (glucose) of bacteria to lead to a bacteriostatic effect [109]. As an example of the antibacterial activity of hypothiocyanite, a study investigated LPO-synthesized hypothiocyanite, dose-dependently, on certain *S. pneumoniae* strains [38]. In the in vitro system, the antibacterial activity of LPOS was observed based on the counted colony numbers. Additionally, a second experiment was performed to identify the antibacterial agent by adding H_2_O_2_ scavenger catalase. The action of catalase removed the excess amount of H_2_O_2_, and the product of the system, hypothiocyanite. With the obtained results, the antibacterial activity directly correlated with LPOS product hypothiocyanite. Several variables based on the *Streptococcus* were also performed, such as a comparison of hypothiocyanite activity against encapsulated and capsule-free mutants. Still, these details are not discussed for the scope of the review.

The LPO system (LPOS)’s antibacterial activity was tested in different studies as well. For instance, bovine milk isolated LPOS was tested against *E*. *coli* to investigate the change in the antibacterial activity of the protein against lactose-reduced milk whey [110]. LPOS-treated *E. coli* population decreased after 4 h, indicating the antibacterial activity of LPO. The solo LPOS-treated group was selected as a control group, and different versions of milk were tested to identify the antibacterial activity levels. As the main point of the research, the lactose-reduced whey showed the highest activity among other variants of milk and whey. This result adds a different perspective in both terms of application and improvement methods of LPOS in the antibacterial area. The antibacterial application of LPO was also investigated with the nanoparticle method. Silica nanoparticles were combined with bovine milk LPO and tested on antibiotic-resistant bacteria, such as *E. coli Salmonell typhii*, and *Streptococcus* sp. [111]. The in vitro study showed a significant inhibition rate between 94 and 96% for used bacteria. Similar research was conducted with silver nanoparticles combined with LPO [39]. The effect of nanoparticles against the antibacterial activity of LPO was tested on *E. coli*. The experiment results showed antibacterial activity of both LPO and LPO nanoparticles, with more sustainability observed on nanoparticle LPO in terms of bacterial concentration increase over time.

Since the LPOS is based on the presence of H_2_O_2_ for the oxidation reaction, LPO itself can be combined with different types of molecules. For instance, the antibacterial role of Lf and LPO, isolated from camel colostrum, were investigated against *Acinetobacter baumannii* in both in vitro and in vivo mice models Table 2 [112]. In vitro study confirmed the antibacterial activity of both proteins, with LPO having the higher activity against the *A. baumannii*. Samples of lung and blood were taken from Infected mice to observe bacterial concentrations. Combined treatment of Lf and LPO successfully decreased lung bacterial numbers and prevented transmission of bacteria via blood. According to the researchers, the results show the synergic interaction between Lf and LPO in antibacterial activity.

One point that needs to be mentioned is the combined application of LPO with XO, as it was discussed based on research in the XO section. To briefly re-mention, the emphasized part was the existence of XO substrates in the neonatal saliva and induced antibacterial activity with a milk-saliva combination (Figure 3). In terms of LPOS, the production of H_2_O_2_ is mediated by specific oral bacteria in adults; thus, this way, the LPOS system can be activated in the adult saliva [15]. On the other hand, the oral microbiome of infants is less diverse than that of adults [113]. This connection might be another indicator of XO being a source of H_2_O_2_ production for LPOS activation, an evolutionary characteristic of lactation of mammals. A study might be shown as supportive data that showed the increased concentrations of XO and XDH in lactating rats [114]. Nevertheless, there is no doubt that lactation is a natural platform that creates the opportunity for antibacterial activity by LPO-XO. Yet, direct research that aims to investigate the antibacterial efficiency of this system in recent years is deficient. Studying this system not only holds the potential to discover new aspects of antibacterial activity but also possesses a new perspective on the mother–infant relationship in terms of lactation and microbiome influence.

### 2.5. Immunoglobulins

**Figure 4:**
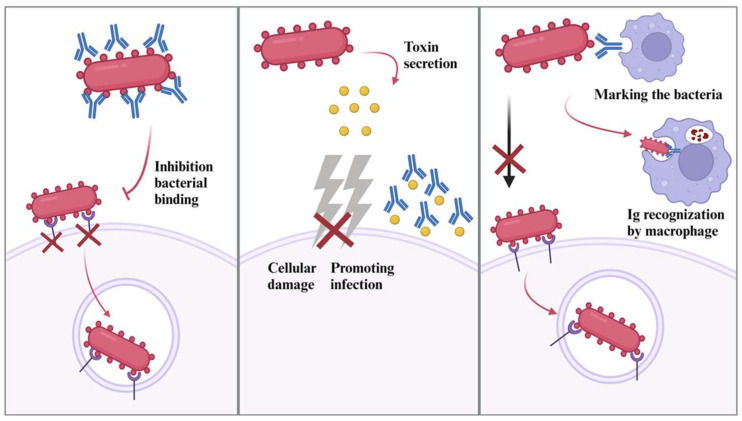
Antibacterial mechanisms of immunoglobulins. Igs can show three main mechanisms during bacterial infection. First, Igs can directly bind to the bacteria and inhibit host cell binding. Secondly, secreted toxins by the bacteria during the infection can be blocked by Igs to prevent cellular damage and host cells’ tendency to infection. Lastly, Igs can bind into bacteria and initiate macrophage recognition by regulating antibody receptor interaction with bacteria and macrophage. The interaction leads to phagocytosis of the bacteria, thus preventing their cellular interaction for infection [117].

Igs are glycoproteins that are known for their role in the immune system, equipped with multifunctional protective activities, specifically binding into antigens on viruses and bacteria [115]. This binding capability is mediated by two antigen-binding sites, including antigen-binding fragments [116]. Igs can show diverse mechanisms against toxins and pathogens, but in terms of bacterial infection, the main mechanisms of Igs directly bind into the bacteria to lead cell lysis of bacteria by penetrating the membrane, binding and marking the viral particles for phagocytosis recognition, and partially neutralization of the bacterial secreted toxins and disrupt the cell entry of bacteria (Figure 4) [117]. The composition and concentration of Igs differ based on the type of milk. For instance, a specific type of IgA (secretory) is mainly found in human milk, and its unique structure enables certain activities such as mucosal defense, inflammation level controlling, and antipathogenic activities [118]. On the other hand, IgG is the main Ig type (IgG1 and IgG2) found in bovine colostrum and milk, which is also capable of binding into pathogens, including bacteria [115].

As an example, a study investigated the interaction between both human and bovine IgA with bacteria in terms of immune interactions [119]. The study showed the non-specific binding of these antibodies (no significant difference in binding rate between two types of IgA, except for one species of *E. coli*) to commensal (at high rates, 78 to 94%) pathogenic (low and high, 24 to 83%), and probiotic (low and high, 17 to 80%). Some of the used species of bacteria are some species of *Lactobacillus* for the prebiotic class, *Salmonella typhimurium*, *Yersinia enterocolitica*, and some species of *E. coli* for the pathogenic class, and some species of *Bacteroides* and *Ruminococcus obeum* for commensal class were used. The potential mechanisms behind these interactions are Ig’s directly binding capability to bacteria, and potentially marking them (especially pathogenic bacteria) for phagocytosis. Additively, glycans that comprise IgA play a major role in the binding of bacterial components [120]. Since glycosylation impacts the function of IgA, especially its binding into bacteria [121], variations among different host Igs in antibacterial activity are expected. This characteristic of IgA can lead to multiple outcomes, especially in the gut region. An in vivo study demonstrated that IgA can identify gut bacteria that possess the potential to lead to inflammation among the microbiota [122]. The interaction of IgA with the intestinal microbiota of mice and humans was investigated. The findings indicated that IgA could specifically bind disease-causing potential carrier bacteria in the mice gut microbiota and potentially identify the tendency of inflammation marks.

IgG can also act as a regulator during gut infection with antibacterial activity. A study researched the activity of gut IgG in systemic infection [123]. At first, mice feces were analyzed to determine if there were sufficient amounts of Igs (IgG, IgA, and IgM) in pathogen-free microbiota. The results indicated a reasonable amount of Igs in pathogen-free wild-type mice. Thereafter, the activity of pre-existing IgG was analyzed by inducing the transmission of bacteria from the gut by disrupting epithelium to blood in mice. Fecal analysis showed an increase in the amounts of IgA and IgG, specific to symbiotic bacteria. The following results after one week showed significantly increased numbers of IgG-bound fecal bacteria, potentially indicating the IgG’s mechanism of action during the disturbance in the epithelium in the gut. Two main bacteria, *E. coli*, and *Escherichia fergusonii*, were found that are majorly responsible for causing the death of mice in induced infection. There are differences between IgG and IgA, maybe not mainly by mechanism, but by the environmental conditions that they are prone to show activity. Studies that primarily focus on IgG activity in similar cases are relatively deficient, especially when compared with IgA.

Lastly, to highlight an important point, IgA antibody levels were investigated from breast milk against four types of streptococcal species, which can be found in oral microbiota and cause diseases [124]. The obtained human colostrum samples showed significant levels in terms of IgA concentration levels, followed by IgM and IgG, respectively. The study indicated all tested samples contained sufficient levels of antigen-specific IgAs for streptococcal species and their virulence antigens. Such a conclusion was also discussed in the XO section; these results indicate the interaction of breast milk and infant oral microbiome and that components of breast milk cause antibacterial activity on the first contact. The current data indicate that the antibacterial activity of Igs is mediated via indirect pathways, such as initiating immune responses or exposing the pathogenic microorganism by binding into it, rather than a direct neutralization. This feature is also one of the main reasons that make Igs a promising alternative in antibiotic treatments. Since Igs administration can induce immune responses and host defense, it is possible to reduce the use of antibiotics in certain areas with Ig treatment. Such conditions were discussed in a review [125] indicating the potential of Igs in animal production to decrease antibiotic application, especially by possessing a significantly lower risk of causing a new resistance development to targeted bacteria. A similar study tested LZ as an alternative to antibiotics in nursery pigs [126]. In terms of growth and gastrointestinal health, antibiotics (carbadox/copper sulfate) and LZ showed significantly close results, which created the potential of LZ as an alternative in this related area. These characteristics might explain why these milk antibodies are shaped to regulate the activity of gut pathogenic microorganisms in the early stages of growth [127].

### 2.6. Oligosaccharides

Carbohydrates with three to ten monosaccharide residues covalently bound by glycosidic linkages are known as OS. But other than lactose, disaccharides—which have just two residues—are frequently referred to as OS as well. The two major categories of OS are neutral and acidic. The residues of charged carbohydrates are absent from neutral OS. Nevertheless, N-acetylneuraminic acid (sialic acid) residues, which are negatively charged, are present in one or more acidic OS (hence the term acidic). In contrast to goat, cow, and sheep milk, which have OS contents and compositions of less than 0.3 g/L, human milk has OS contents of more than 12 g/L. In comparison to cow and sheep milk, goat milk also contains a larger quantity of acidic and neutral OS [128].

The past ten years have seen significant advancements in food technology, chemical sciences, and chemical-enzymatic synthesis for large-scale manufacturing, which have all contributed to the increased interest in milk OS among scientists [129,130]. Given the vast quantity of milk produced by cows, goats, and other animals, even if the quantity and number of OS in animal milk are relatively small in comparison to those in human milk, it could be worthwhile to isolate a few constituents [131]. The highly concentrated OS found in human milk was the initial focus of most investigations on milk OS functionality. Up to 200 structures have been identified in thorough investigations that have characterized HMOs in detail [132,133]. In order to find the similarities in the structures and bioactivities of milk OS from different mammalian species, this field of study has lately grown. Research into the potential medical benefits of bovine milk oligosaccharides (BMOs) and the mostly underutilized BMO-containing side streams in the dairy industry has been spurred by the industrial production of bovine milk [134].

**Figure 5:**
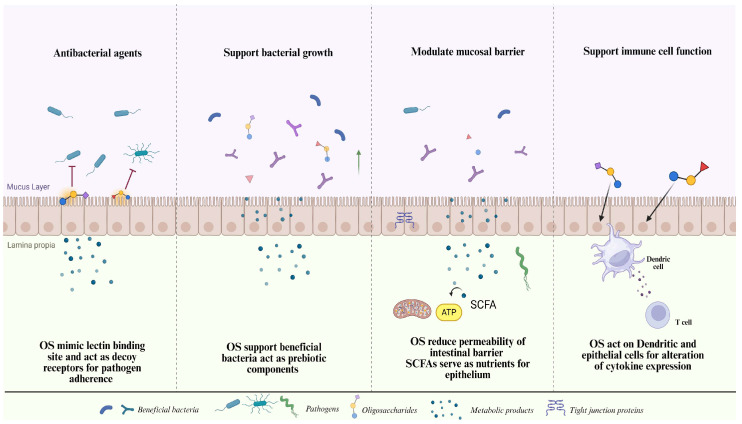
A schematic overview describing the different mechanisms of actions of milk OS to overcome bacterial infections. OS acts as an antimicrobial agent by attaching to toxins and pathogens and by directly interacting with epithelial receptors to stop invasive pathogen adherence and infection. OS serve as prebiotics, favorably promoting bifidobacteria and other intestinal flora. Additionally, protecting against infectious diseases is this selective advantage that bifidobacterial species have over pathogens. The metabolites generated during OS fermentation play a role in the physiology of neonatal development. The primary metabolites of OS fermentation, short-chain fatty acids, affect how intestinal epithelial cells mature. By controlling the production of tight junction proteins and lowering intestinal permeability, OS improves barrier function. OS also changes how proteins are expressed in the mucus and glycocalyx layers. OS contributes to the preservation of immunological homeostasis by interacting with immune cells (DCs, T cells, and B cells) and influencing the expression of pro- and anti-inflammatory cytokines [103,138].

Certain OS possesses vital physiological characteristics that enhance human well-being, such as impeding pathogen growth and replication, enhancing the intestinal milieu, diminishing the likelihood of cardiovascular ailments, and functioning as a sugar alternative. OS are also thought to function as prebiotics to encourage the growth of gut probiotics [135]. Additionally, the composition of HMOs is highly influenced by the mother’s genetic composition. Not only genetics, but other external conditions, such as age and diet of the parent also affect the structure of HMOs [136]. This fact is mostly mediated by the milk microbiome and significantly influences the development of the infant’s intestinal microbiota. Similar to the XO-LPO system, protection of the infant, including against bacteria, is initiated by obtaining HMOs during lactation. Because of their functional and vital roles, they are involved in the development of functional food, medical components, and other health-supportive products (Figure 5) [137].

The antibacterial and prebiotic properties of OS have been investigated in a variety of contexts. Their outstanding broad-spectrum antibacterial capabilities are mostly due to numerous synergistic actions, such as pathogen cell wall breakdown. Furthermore, OS might prevent harmful substances from playing a part and prevent harmful microorganisms from adhering. Probiotic microbial growth has also been shown to be stimulated by natural OS [33,103].

Research by Asadpoor et al. [139], for instance, showed that eight bacteriostatic, non-digestible OS efficiently prevent the adherence of foodborne bacteria. The presence of galacto-oligosaccharides (GOSs) in lactic acid bacteria from fermented milk-permeated beverages was found to vary in concentration from 8.7 to 26.8 mg/100 mL sample. These GOSs showed a noteworthy inhibitory impact on more than half of the tested microorganisms [140].

As one of the most common causes of diarrhea globally, *C. jejuni* is an example of a pathogenic bacteria that is suppressed by OS from human milk [141]. A further recent study discovered that 2′-FL 80% lowers *Campylobacter* penetration in addition to inhibiting binding to the mucosal surface [142]. The results of these investigations are consistent with a prospective study of ninety-three mother–infant dyads who were nursing. The concentrations of α1-2 fucosylated HMOs in the mother’s milk were found to be inversely correlated with an infant’s susceptibility to diarrhea caused by *C. jejuni*. Based on these results, researchers determined that key components of human milk that help protect against contagious viruses include α1-2 fucosyl OS [143].

The potentially harmful effects of AMR infections are especially dangerous for infants [144]. There is evidence to show that enteric AMR pathogens may colonize or infect young children at an early age [145]. A high HMO concentration in the early infancy gut may lessen children’s susceptibility to AMR infections colonizing their guts. Invading AMR pathogens may be immediately neutralized by HMOs, and they may also lessen gut inflammation, which otherwise creates an environment that is conducive to Enterobacteriaceae development and antibiotic resistance genes (ARGs) transmission. Throughout the first two months of life, *B. infantis*, which grows in gut environments with high HMO levels, has also been demonstrated to reduce the generation of proinflammatory cytokines and is linked to lower quantities of fecal calprotectin, a hallmark of intestinal inflammation [146,147]. One of the most important ways to stop the emergence of dangerous AMR infections, such as AMR Enterobacteriaceae, is to reduce gut inflammation in the gut environments of breastfed infants and HMOs [148].

Given the possible advantages listed above, breastfeeding should be further investigated as a scalable and reasonably priced approach to combating antibiotic resistance. Reducing early-life antibiotic use and boosting gut colonization resistance to AMR infections, which are becoming more prevalent in the community, are two potential benefits of optimizing breastfeeding habits in high-income nations, where less than half of newborns receive any human milk at six months of age. Accelerating measures to promote breastfeeding at the policy level would be necessary if breastfeeding or components of human milk were found to be protective against colonization or infection with AMR bacteria [149]. Extracellular vesicles (EVs), maternal antibodies, HMOs, antimicrobial peptides, and the milk microbiota are just a few of the human milk components that may protect against early-life colonization by AMR pathogens and ARGs. These emerging mechanistic pathways should be further investigated as novel targets for intervention and, in cases where optimal breastfeeding practices are not feasible, supplementation. In the global effort to address AMR, breastfeeding and human milk supplementation deserve more attention as potential preventive interventions [148].

Furthermore, serving to guard against *E. coli* pathogenesis is the OS found in human milk. Serious diarrheal illness with elevated infant death rates is caused by Enteropathogenic *E. coli* (EPEC). Compared to control or GOS-treated pups, HMO-treated mice show considerably less EPEC infection, and pre-incubating EPEC with combined HMO fractions lessens the pathogenic colonization of cultured epithelial cells [150]. These findings concur with those of Coppa et al.’s study [151], which showed that acidic HMO fractions reduced the infectivity of *E. coli* serotype O119. Conversely, sialylated structures like 3′-SL show significant suppression of *Helicobacter pylori* adherence to gastrointestinal epithelial cells [152]. Moreover, enteropathogenic *Salmonella*’s adherence and contagiousness are significantly decreased by fucosylated HMO structures [153].

Furthermore, it was shown that OS produced from bovine colostrum may prevent EPEC *E. coli*, *C. sakazakii*, and *S. enterica* [154]. It was also demonstrated that OS, both neutral and acidic, extracted from bovine colostrum prevented *S. enterica* IID604 from adhering to Caco-2 cells [155]. HMOs demonstrated antibacterial and antibiofilm activity against GBS, antibiofilm activity against a methicillin-resistant strain of *S. aureus*, and antimicrobial activity against a strain of *Actinobacter baumannii* in a different investigation using growth and biofilm assays [156]. By preventing bacterial growth and the development of biofilms, Spicer et al. [157] also showed how HMOs with bacteriostatic qualities have arisen as an alternate treatment approach against antibiotic resistance. In addition, a research team has emphasized the usage of HMOs as an alternative to antibiotics and performed multiple experiments to show the antibacterial activity against *Streptococcus agalactiae* [157]. It also has been mentioned that HMOs can be combined with certain types of antibiotics against *S. agalactiae* as well [158]. Furthermore, compared to native BMOs, fucosylated BMOs more successfully increased the antiadhesive activity of Caco-2 cells [159]. As for antibacterial activity, Craft et al. [160] discovered that fucose residue placement and quantity on HMOs are crucial. Therefore, because of their strong antibacterial activity, OS offers a wealth of research opportunities.

## 3. Other Antibacterial Components

### 3.1. α-Lactalbumin

α-Lactalbumin (α-La) is a predominant whey milk protein and one of the two regulatory components that are actively involved in lactose synthase in mammary epithelial cells. It also possesses an important role in many biochemical and nutritional studies focused on early infant development [33]. During lactation, human milk has approximately 2.44 ± 0.64 g/L mean concentration of α-La [161], containing 22% of total protein and 36% of overall whey proteins. Thereafter, bovine milk α-La is the second most abundant protein, accounting for 3.5% of total protein and nearly 17% of whey proteins [162].

In addition to the role of α-La in lactose biosynthesis and infant development, some studies are also emphasizing the antibacterial activity. The research investigated the antioxidant and antibacterial activity of α-La, isolated from camel and bovine, in both apo and holo forms [163]. Several types of bacteria, and in addition, some types of fungi, were used during the experiment. In terms of antibacterial activity, apo camel α-La managed to show its antibacterial activity against one of four bacteria, *P. aeruginosa*; meanwhile, it was ineffective against *S. aureus*, *E. faecalis*, and *E. coli*, just like in holo and apo bovine and holo camel α-La. Interestingly, a recent study showed that a complex formed between α-La and Car showed increased antibacterial activity when compared with only Car activity [164]. The study first exemplified the molecular interaction between Car and α-La. Then, the complex was tested on *E. coli* and *S. aureus* and successfully showed antibacterial activity in tests when compared with solo Car treatment. As a result, the potential of α-La as an antibacterial enhancer was also emphasized.

### 3.2. Epidermal Growth Factor

EGF is a protein known for the proliferation of various types of cells, such as fibroblasts and epithelial cells, as a mitogenic factor [165]. The biological action of the EGF is mediated via interaction with the EGF receptor (EGFR) to initiate intracellular signaling [165]. EGF is found in diverse body fluids: saliva, urine, plasma, intestinal fluid, amniotic fluid, and particularly in milk [166]. It is typically present in human milk at concentrations varying between 0.038 and 0.045 ng/mL [167], which may increase to approximately 100 ng/mL following parturition [168]. EGF, as an essential component of both human colostrum and milk, plays an essential role in promoting the growth and repair of the gastrointestinal tract of the newborn. Even though it is limited, there are some studies investigating the contribution of EGF to antibacterial activity.

The role of EGF and activation of EGF receptors (EGFR) were investigated from the perspective of transmission of pathogenic *E. coli* that accumulated in the gut, causing late-onset neonatal sepsis [169]. Late-onset neonatal sepsis occurs in neonates after three days of birth, commonly when Gram-negative pathogenic bacteria cause infection and are observed in the bloodstream of the neonate [170]. During the study, the role of EGFR was first tested on mice, as the *E*. *coli* colonization killed the mice when the EGFR inhibitor was also administered. The following experiment identified the location of the *E. coli* colonization in the lamina propria of the colon. Normally, goblet cells form the goblet cell-associated antigen passages to increase the efficiency of immune response and allow the antigens to be delivered to immune cells [171]. As expected, the immune responses are yet to be strong enough and have immunological memory to fight off an upcoming infection. Consequently, the discussed study found that EGF from early lactation interacts with EGF receptors and decreases the formation of goblin cell passages that protect against upcoming infections, especially on pathogenic *E. coli*, in early life. The role of EGF in the intestine area came out during the first days of lactation. As it prevents further fatal infection and bacterial spread, EGF’s role can be registered as an antibacterial activity.

### 3.3. Glycomacropeptide

Glycomacropeptide (GMP), which is also known as caseinomacropeptide, is a bioactive milk peptide that is liberated from κ-casein via either physiological enzymatic digestion or chymosin digestion while manufacturing cheese [172]. In the adult human gastrointestinal (GI) tract, pepsin hydrolysis produces GMP following milk consumption [173]. The bovine GMP is one of the most studied and well-characterized milk proteins because of its wide commercial availability. The most well-characterized ones that have a beneficial effect on human health are those that have antibacterial, prebiotic, remineralizing, digestive and metabolism-regulating, anti-tumor, and immune-modulatory properties [174].

GMP, a highly sialylated glycoprotein, and other carbohydrates together determine the peptide’s antibacterial activity, which is mostly a decoy receptor impact [175]. The cholera toxin (CT) and casein whey GMP interacted directly to block CT from binding to its target receptor, ganglioside GM1, which is present in the CHO-K1 cell line. The inhibition of CT binding to GM1 was lessened by the enzymatic breakdown of GMP using sialidase and pepsin. Therefore, the peptide sequence and the presence of sialic acid may be responsible for GMP’s direct inhibitory impact on CT [175]. A later investigation revealed that GMP had a dose-dependent binding capacity to harmful bacteria, such as *Salmonella enteritidis*, *Morganella morganii*, and enterohemorrhagic *E. coli* (EHEC O157), but not to *Lactobacillus casei*, a probiotic. According to the study, asialo-GMP was partially reduced, and peroxidation entirely abolished GMP’s attachment to *S. enteritidis*, while desialylation and peroxidation inhibited its binding to EHEC O157. This indicates that different carbohydrate molecules, such as sialic acid, may facilitate GMP binding depending on the bacteria [176].

Subsequent research added to our understanding of the bacteria, both pathogenic and probiotic, whose adherence is hindered by GMP. With regard to probiotic bacteria, GMP inhibited *L. acidophilus*, *Lactobacillus pentosus*, and *L. casei* from binding to HT-29 cells [177]. Brück et al. [178] investigated a number of distinct digests of bovine GMP to inhibit *Shigella flexneri*, *S. typhimurium*, and EPEC *E. coli* from adhering to Caco-2 cells by directly attaching the milk component to the pathogens. For EPEC, pepsin/pancreatin and pepsin-digested GMP were the most efficient combinations. GMP that had been pepsin or pancreatin-digested more strongly inhibited *S. typhimurium*, but undigested or pepsin-digested GMP had the greatest effect on *S. flexneri*. The sialic acid substructure of GMP, which largely retains its structure after digestion, was thought to be the reason for its capacity to maintain a decoy receptor function.

### 3.4. Glycosaminoglycans

Glycosaminoglycans (GAGs) are long-chained, negatively charged polysaccharides, which are usually covalently attached to proteins, resulting in the formation of proteoglycans [179]. They can be found as part of the extracellular matrix or directly located on the cellular membrane. GAGs are classified into four types based on the core disaccharide units: heparin/heparan sulfate, chondroitin sulfate, keratan sulfate, and hyaluronic acid [180]. The different conformations of GAGs contribute to their involvement in numerous biological functions, such as immunity development, cellular signaling, and pathogenesis [181]. Additionally, GAGs can serve as main targets in the initiation of viral infections.

In particular, GAGs isolated from milk can show significant activity as anti-infective agents [182]. The levels of GAGs in human milk change throughout lactation. The highest level of concentration is observed on day 4 at 3.8 g/L. The concentration declined to 0.4 g/L after one month, representing a 73% reduction. This decline occurs between days 4 and 10, highlighting the significance of GAGs in the first two weeks of life [33]. A study was conducted to observe the anti-infective activity of GAGs derived from human milk against pathogenic bacteria, *E. coli*, and *Salmonella fyris.* During the experiments, GAGs were isolated from different milk samples to observe their antibacterial activity. Results showed that the purified human GAGs complex inhibited, *in vitro*, the adhesion of *E. coli* and *S. fyris* to human intestinal cells [183].

## 4. Limitations, Advantages, and Disadvantages of Milk and Colostrum Compound Development and Usage

Although these compounds possess significant antibacterial activity and show considerable potential in antibacterial treatment, when the antibacterial activity of milk and colostrum aimed to be administered as a compound, alongside advantages some limitations as well became apparent.

For instance, when the mechanism of XO is thought of, its antibacterial activity is mediated by hydrogen peroxidase synthesis. Conversion of XOR to XO is highly regulated during the inflammation. During the first phases of the infection. activation of XO develops antibacterial activity by ROS, but sustained activation can create excess amounts of ROS and increase inflammation [184]. In terms of application, decided dose amounts play a crucial role in obtaining maximum efficiency while decreasing the potential negative feedback. Synergistic administration would increase efficiency and decrease the risk of potential ROS-derived damage. Using XO to procure the needed hydrogen peroxidase for LPOS directly meets this problem, as most of the created ROS will be utilized by the LPOS. Since sole treatment of LPO will be insufficient in terms of having a significant antibacterial activity, the combination of these two molecules fixes the negative aspect of both molecules.

The composition of HMOs is significantly suitable for prebiotic application and treatments in gastrointestinal regions. HMOs are not only resistant to metabolic enzymes but also remain unaffected by stomach acid and gastrointestinal absorption [185]. This gives HMOs a huge advantage in their therapeutic application in certain diseases and supplements as prebiotics. Yet, one major challenge that is discussed in terms of HMO production is shaping the biological function [136]. Since genetic factors are the main factor that influences the molecular structure of the HMOs, the relationship between the structure and function needs to be recorded specifically. Insufficiency in the current data about the relationship between HMOs and milk microbiome is an addition to this deficiency [186]. This necessity hinders the production and administration of HMOs in terms of therapeutic application. Additionally, the complexity of HMO structures alongside insufficient availability and the high cost of isolating and synthesizing them can be thought of as the main limitations [129]. These factors, collectively, lead to significant restrictions in HMO research and broader application, highlighting the need for innovative approaches to overcome these limitations.

Igs have a unique spot in therapeutic application since they can also be used to develop immune responses to deal with bacterial infection indirectly. Some types of Igs are potent in certain areas in terms of therapeutic application. In this way, Ig’s range of application is wider than most other compounds. Administration of Igs has several routes of administration and some of them can lead to adverse reactions [187]. Even though most of these reactions can be minimized, long-term therapies can cause abnormalities such as inhibition of endogenous Ig production, which is reversible after some time without treatment. One of the most discussed routes of administration of Igs for its high ratio of adverse effects is the intravenous route. A review discussed the adverse effects of Ig therapy in detail [188]. Even though most of these adverse effects seem likely to rarely show up, still some of the adverse effects can be observed more frequently as neurologic, hematologic, or dermatological reactions. Especially if the Ig therapy is desired to be used for increasing the host’s overall immune system for infections, the administration phase lasts for months. To decrease these side effects in the long term, shaping the treatment specific to the individual is highly necessary.

LZ exerts its antibacterial activity by degrading bacterial cell walls. However, a disadvantage is that it does not show the same efficiency against all types of bacteria. Specifically, the antibacterial efficiency of LZ remains insufficient against the cell walls of Gram-negative bacteria when compared to Gram-positive [189]. Consequently, it hinders the application of LZ and restricts its effectiveness against a wide range of bacterial infections. From another perspective, the ability of LZ to be used in combination with other antibacterial molecules might be considered as a significant advantage. An in vitro study [73] established that LZ could act together with Lf to show bactericidal activity. Experiments conducted on *E. coli*, *Salmonella Typhimurium*, and *V. Cholerae* revealed Lf significantly enhances the activity of LZ. The process begins with Lf rupturing the cell membrane of the Gram-negative bacteria by binding to the lipopolysaccharides on the outer cell membrane. This action allows LZ to penetrate more proficiently to break down the bacterial cell wall, indicating its effectiveness could be improved in a combined treatment.

Lf’s antibacterial activity has been shown against a broad range of bacteria, and it has long been understood to play a critical role in host defense systems [190]. The issue with bacteria generally is that lf can only be used in conjunction with antibacterial medications against pathogens that are unable to use Lf as an iron source [191]. Yet, certain drugs that do not show this disadvantage can cause toxic-based adverse effects and increase the ratio of drug-resistant microorganisms at the site of treatment. At this point, Lf not only differs from drugs as a natural compound with non-toxic characteristics but also can demonstrate unique mechanisms that cannot be observed from traditional drugs [192].

Nevertheless, these limitations are not only affecting the production and therapeutic application of these compounds. Some of these compounds are insufficiently investigated in current literature, especially when compared to other antibacterial compounds. For instance, research on Lf and HMOs is at a significant level in many perspectives. For other compounds, however, like XO and LPO, the studies that directly investigate their antibacterial activity are very few. Moreover, the XO-LPO system is one of the major antibacterial compounds that directly indicates the evolutionary relationship between the infant and mother. Still, just a few studies successfully mentioned this relationship, and experiments to explain it in detail. Potentially, the needed ethical approvals in the background are needed to study components of human colostrum is also a hindering situation. Igs and HMOs show significant differences based on the type of colostrum and the genetic background of the mother. This condition not only generates difficulties in the therapeutic application and production but also creates gaps in the literature. It is not hard to point out the power of these compounds in antibacterial research, as this review intended to. Yet, we cannot fully demonstrate that these compounds are or should be the preferred agents in related infections.

## 5. Discussion and Conclusions

One possible approach to solving the problems caused by the increasing number of antibiotic resistance genes and the growing concern about chemical preservatives is research into the antibacterial properties of milk and colostrum. Natural defense substances such as Lf, LZ, LPO, Igs, OS, etc., represent a potential source of biotherapeutic agents. In this article, it is shown that these components are crucial for the development of the immune system of the offspring, as they accelerate the maturation of the immune system and, most importantly, have significant antibacterial properties.

A thorough study of the antibacterial properties of milk and colostrum shows that they are very promising for the treatment and prevention of a variety of diseases caused by microbes. It should be emphasized that certain types of antibacterial milk proteins tend to be associated with infections in different regions and environmental conditions and interact with specific molecules. This not only provides an opportunity to advance alternative therapeutic modalities but also addresses the urgent need for new techniques to combat antibiotic resistance and adds promising insights to the field of research.

Another unique consideration when looking at the antibacterial case is the ability of these proteins to exert their activity early in life. When looking at evolutionary aspects, particularly in lactation, it can be observed that some of these molecules naturally form complexes and/or systems to express antibacterial activity and specify it in terms of time and location. Further investigation into the exact processes by which these components of colostrum and milk exert their antibacterial properties, as well as possible synergistic effects, will contribute to our understanding. A potential and comprehensive strategy to promote ecosystem health and human well-being in addressing the problem of antibiotic resistance and the search for sustainable alternatives is the thorough exploration of milk and colostrum as therapeutic agents.

## Figures and Tables

**Table 1:** Antibacterial compounds, their levels, and properties in human and bovine colostrum and milk.

Component	Human		Bovine		Properties
	Milk	Colostrum	Milk	Colostrum	
Lactoferrin [6,9,12,13]	1.5 g/L	29.85 g/L	0.02–0.75 g/L	1.5–5 g/L	Antimicrobial activity, anti-inflammatory action, regulation of cell growth, immune regulation, ROS scavenging activity
Immunoglobulins [6,10,12,14]	1.3 g/L (IgA, IgM, IgE, IgG)	IgG 27.9 ± 23.2 g/L SIgA 16.4 ± 6.1 mg/L	IgG1 0.31.0.40 g/L	IgG1 34–87 g/L	Immune booster, regulation, protection
			IgG2 0.03–0.08 g/L	IgG2 1.6–6 g/L	Antimicrobial activity, pathogen recognition
			IgA 0.04–0.06 g/L	IgA 3.2–6.2 g/L	
			IgM 0.03–0.06 g/L	IgM 3.7–6.1 g/L	
Lactoperoxidase [6,10,15]	0.89 mU/mL	3.28 mU/mL	13–30 mg/L	11–45 mg/L	Antibacterial activity with systematic composition, combined activity with XO, Lf, and Ig
Lysozyme [6,9,16,17]	200–400 µg/mL	0.37 mg/mL	0.05–0.22 µg/mL	0.14–0.7 mg/L	Antimicrobial activity, complementary interaction with Lf and Igs, neuroprotection
Xanthine oxidase [15]	0.52–0.91 mU/mL	8 mU/mL	35 mg/L	-	Antibacterial activity with ROS synthesis, synergic interaction with LPO
Oligosaccharides [9,18,19]	12–13 g/L	22–24 g/L	0.1–0.2 g/L	0.7–1.2 g/L	Antimicrobial, prebiotic activity, support immune/intestinal system, and brain development

**Table 2:** In vivo studies of milk antibacterial compounds.

Antibacterial Molecule	Result	Reference
CAMP211-225 peptide	Antibacterial activity against antibiotic-resistant *S. aureus*, *E. coli*, and *Yersinia enterocolitica*.	[36]
Lactalbumin	Antagonistic effects against *E. coli* O127 and reduction in diarrhea incidences.	[36]
Lysozyme	An increase in beneficial gut microbial diversity has been observed.	[37]
Lactoperoxidase	LPO-generated hypothiocyanite exhibited antibacterial activity against various Gram-positive and Gram-negative bacteria, and its effectiveness increased in reduced-lactose milk whey.	[38]
Lactoperoxidase	LPO synergically showed antibacterial activity with Lf against drug-resistant *Acinetobacter baumanniii* in mice models.	[39]
Lysozyme	Levels of *Bacteroidetes*, *Bifidobacteriaceae*, and *Lactobacillaceae* had been increased. Reduction in *Firmicutes*, *Mycobacteriaceae*, *Streptococcaceae*, and *Campylobacter* was observed.	[40]
Lysozyme	Increased levels of *Lactobacillus* and mucosal IgA responses had been observed. Faster recovery, lower morbidity, and less mortality from ETEC infection were also noted.	[41]
Lysozyme	Improvement in weaning weight, intestinal health, and levels of *Lactobacillus* had been observed in the group fed with 1.0 g/kg LZ for 14 days.	[42]
Lactoferrin	Lf exhibits antimicrobial properties against both Gram-positive and Gram-negative bacteria, including *E. coli* O157:H7. Its antimicrobial mechanisms comprise bacteriostatic, bactericidal, and anti-adhesion effects.	[43]
Lactoferrin	After four injections, complete eradication of *S. aureus* had not yet been achieved; however, viable bacterial counts demonstrated a two-log decrease following treatments with Lf and/or penicillin G.	[44]
Lactoferricin	Bactericidal activity against *S*. *aureus* and *Pseudomonas aeruginosa* strains was observed with lactoferricin, showing a minimum inhibitory concentration of 1.0–2.0 μg/mL for *S. aureus* and 4.0–8.0 μg/mL for *Pseudomonas aeruginosa*.	[45]
Lactoferrampin	Lactoferrampin displayed a wide-ranging antibacterial efficacy against various bacterial strains; however, *Porphyromonas gingivalis*, *Actinomyces naeslundii*, *Streptococcus mutans*, and *Streptococcus sanguis* exhibited resistance to this peptide.	[46]
Lactoferricin	Bactericidal activity against *E. coli* and *E. faecalis* strains was observed with lactoferricin, exhibiting a minimum inhibitory concentration of 0.5–1.0 μg/mL for *E. coli* and 2.0–4.0 μg/mL for *E. faecalis*.	[47]
Lactoperoxidase	Decreases in both Gram-positive and Gram-negative bacteria levels, notably *E. coli* and *Pseudomonas* species, occur with the addition of external hydrogen peroxide supplementation.	[48]
Immunoglobulin	The IgY protected fully inhibited diarrhea induced by enterotoxigenic *E. coli* in challenged piglets.	[49]
β-lactoglobulin	Bovine β-lactoglobulin displayed growth inhibition against *S*. *aureus*; however, it did not exhibit effectiveness against *E. coli*. Moreover, it demonstrated inhibitory activity against *Streptococcus uberis*.	[50]
